# Correlation between Tick Density and Pathogen Endemicity, New Hampshire

**DOI:** 10.3201/eid1504.080940

**Published:** 2009-04

**Authors:** Seth T. Walk, Guang Xu, Jason W. Stull, Stephen M. Rich

**Affiliations:** University of Massachusetts, Amherst, Massachusetts, USA (S.T. Walk, G. Xu, S.M. Rich); New Hampshire Department of Health and Human Services, Concord, New Hampshire, USA (J.W. Stull); University of New Hampshire, Durham, New Hampshire, USA (J.W. Stull)

**Keywords:** Vector-borne infections, Lyme disease, Ixodes spp., ticks, Borrelia burgdorferi, Anaplasma phagocytophilum, Babesia microti, New Hampshire, dispatch

## Abstract

To assess the endemicity of tick-borne pathogens in New Hampshire, we surveyed adult tick vectors. Pathogens were more prevalent in areas of high tick density, suggesting a correlation between tick establishment and pathogen endemicity. Infection rates in ticks correlated with disease frequency in humans.

Along the borders of the northeastern and the upper midwestern United States, black-legged ticks (*Ixodes scapularis*) are invading new areas (*1*–*3*). Because this tick is the principal vector of a number of human pathogens, defining and monitoring its possible expansion are imperative. Little information is available about *I. scapularis* invasions, including the relative rates of pathogen carriage as vectors expand their range and establish locally enzootic cycles. To assess the endemicity of 3 tick-borne pathogens (*Borrelia burgdorferi*, *Anaplasma phagocytophilum*, and *Babesia microti*) throughout New Hampshire, we surveyed adult *I. scapularis* vectors.

## The Study

**Figure 1:**
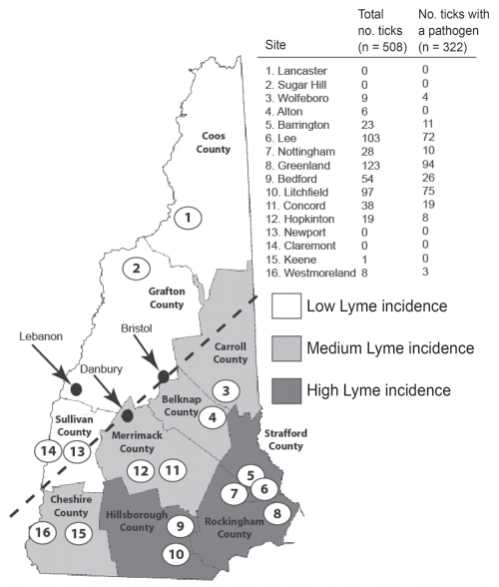
Results of *Ixodes scapularis* tick sampling and pathogen screening in New Hampshire. County names are shown in bold and sampling sites across counties of low (white), medium (light gray), and high (dark gray) Lyme disease incidence are numbered. Dashed line indicates the leading edge of the expanding *I. scapularis* range. Arrows and black dots indicate cities referred to in the discussion. Numbered open circles identify the locations of the 16 sample sites.

During the fall of 2007, we established 16 sampling sites in the 10 counties of New Hampshire (Figure 1). Levels of reported human Lyme disease had varied among the counties in 2006. We categorized each site as high Lyme disease incidence (HLI), medium Lyme disease incidence (MLI), or low Lyme disease incidence (LLI) according to the reported number of Lyme disease cases per 100,000 persons in 2006 (*4*). As in the neighboring states of Massachusetts (Xu et al., unpub. data) and Maine (*5*), in New Hampshire, ticks are most abundant in coastal counties. The HLI sites (Strafford, Rockingham, and Hillsborough counties) were along the coast and had 37–104 reported Lyme disease cases per 100,000 persons; the MLI sites (Carroll, Belknap, Merrimack, and Cheshire counties) bordered the coastal counties and had 10–19 cases per 100,000 persons; and the LLI sites (Coos, Grafton, and Sullivan counties) were the most inland and had 5–12 Lyme disease cases per 100,000 persons.

We collected adult ticks rather than the conventionally sought nymphs (*6*–*8*) because we believe adults best indicate the pathogen pool in an area. Transovarial transmission of *B. burgdorferi* is negligible, and *I. scapularis* ticks acquire infection by feeding on infected hosts as larvae or as nymphs. Because larval ticks feed during late summer and early autumn, they are more likely to feed on migratory animals, such as birds; nymphal ticks feeding in early summer are likely to feed on resident hosts. Hence, adult ticks are more likely to have taken at least 1 resident blood meal from the site of origin, and their *Borrelia* infection rates (and genotype frequencies) are more representative of endemicity.

We visited each site twice within 1 month (October or November 2007) and sampled each site at each visit for ≈25 minutes. A total of 509 adult ticks were collected by drag-sampling vegetation. As expected, ticks were most abundant at HLI sites and moderate at MLI sites; no ticks were found at the 3 LLI sites, including 2 sites (Newport and Claremont) that were close to where ticks have previously been reported (*9*). Likewise, we did not find ticks on 6 deer carcasses at hunter check stations in nearby towns (Newport, Danbury, and Bristol).

Ticks were transported to the laboratory, where they were frozen with liquid nitrogen and pulverized in Eppendorf tubes with plastic pestles (Kontes, Vineland, NJ, USA). DNA was extracted using Epicenter Master Complete DNA & RNA Purification Kits (Epicenter Technologies, Madison, WI, USA). Two duplex real-time–PCR reactions were developed (G. Xu et al., unpub. data) by using oligonucleotide primers and Taqman probes for real-time detection of total tick DNA and *B. burgdorferi* (duplex 1) and *A. phagocytophilum–B. microti* (duplex 2). The most common pathogen found was *B. burgdorferi*, followed by *B. microti* and *A. phagocytophilum* (Table). A total of 322 (63%) ticks carried at least 1 pathogen, and 40 (8%) ticks carried 2 pathogens. The prevalence of ticks positive for both *B. burgdorferi* and *B. microti* was greater than that of ticks positive for both *B. burgdorferi* and *A. phagocytophilum* and accounted for most (78%) coinfections. Neither of the observed coinfections (*B. burgdorferi–A. phagocytophium*, *B. burgdorferi–B. microti*) differed significantly from its expected random occurrence (contingency table analysis p = 0.487, χ^2^ p = 0.926).

**Table:** Pathogen prevalence and coinfection in 2 regions of different tick density, New Hampshire*

Infection	No. ticks collected		
	HLI sites	MLI sites	Total
Single infection			
*Borrelia burgdorferi*	237	29	266
*Anaplasma phagocytophilum*	1	0	1
*Babesia microti*	13	2	15
Multiple infections			
*B. burgdorferi* + *A. phagocytophilum*	9	0	9
*B. burgdorferi* + *B. microti*	28	3	31
*A. phagocytophilum* + *B. microti*	0	0	0
*B. burgdorferi* + *A. phagocytophilum* + *B. microti*	0	0	0

*HLI, high Lyme disease incidence; MLI, medium Lyme disease incidence.

**Figure 2:**
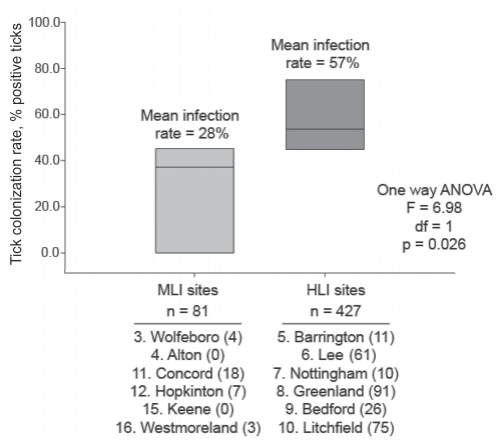
Analysis of variance (ANOVA) of *Borrelia burgdorferi* prevalence in *Ixodes scapularis* ticks isolated from New Hampshire counties of medium (MLI) and high (HLI) incidence of Lyme disease.

We found a significantly greater percentage of *B. burgdorferi*–infected ticks from HLI sites than from MLI sites (Figure 2). *B. burgdorferi* was twice as common in ticks from the HLI sites. Similarly, *B. microti* was more likely to be sampled from HLI sites (5.0%) than from MLI sites (2.7%), although this difference was not significant. Linear regression showed a strong correlation (*R^2^* = 0.90) between the entomologic risk index (total number of ticks × proportion of ticks infected [*6,8*]) in this study and the incidence of human cases of Lyme disease by county in New Hampshire in 2007.

## Conclusions

*I. scapularis* was initially found in New Hampshire in 1985, near the town of Lebanon (*9*). Lebanon is located in a region where we were unable to sample ticks (Figure 1). It is tempting to hypothesize that *I. scapularis* was once more abundant in western counties. In 2001, A.T. Eaton noticed a distribution of ticks similar to what we found (*10*), suggesting that the current distribution has been stable for at least 7 years. Our result that high tick density areas had the highest overall prevalence of pathogens and the highest prevalence of coinfected ticks supports the finding of Hamer et al., who reported a significant difference in the rates of pathogen carriage between recently invaded and *I. scapularis*–endemic areas (*1*). The rates of infection we found are similar to those found by Swanson et al. from 5 other northeastern states (*11*). According to their meta-analysis, ≈40% (± 13%) of 2,109 adult and nymphal ticks were infected with *B. burgdorferi*, 21% (± 17%) with *A. phagocytophilum*, and 9% (± 8%) with either *B. microti* or *B. divergens*.

A noteworthy exception is the prevalence of *A. phagocytophilum*. We detected this pathogen at only 1 site (10 positive ticks from Greenland on 2 independent visits). Human anaplasmosis has been a reportable disease in New Hampshire for at least a decade (*12*), and cases have been rare (0 or 1 reported per year during 1998–2006). However, human anaplasmosis increased substantially in 2007 (3 cases) and 2008 (9 cases).

Human babesiosis appears to be following a similar trend (2 cases in 2005, 3 cases in 2006 and 2007, and 9 cases in 2008), although the disease has been reportable only since 2005. Lastly, we found a strong correlation between entomologic risk index and the incidence of human Lyme disease. This result contrasts starkly with a lack of correlation found by Falco et al. between the abundance of adult female ticks and reported cases of erythema migrans (a common clinical presentation of Lyme disease) in southern New York State (*7*). Improved reporting of Lyme disease by clinicians to state health officials may be responsible for this discrepancy. The data presented here suggest that ERI estimates using adult ticks are accurate proxies for the yearly incidence of human Lyme disease in regions where Lyme disease is endemic.
